# Checklists to detect potential predatory biomedical journals: a systematic review

**DOI:** 10.1186/s12916-020-01566-1

**Published:** 2020-05-18

**Authors:** Samantha Cukier, Lucas Helal, Danielle B. Rice, Justina Pupkaite, Nadera Ahmadzai, Mitchell Wilson, Becky Skidmore, Manoj M. Lalu, David Moher

**Affiliations:** 1 Centre for Journalology, Clinical Epidemiology Program, Ottawa Hospital Research Institute, Ottawa, ON K1H 8L6 Canada; 2 Department of Cellular and Molecular Medicine, Faculty of Medicine, University of Ottawa, Ottawa, ON K1N 6N5 Canada; 3 Knowledge Synthesis Group, Clinical Epidemiology Program, Ottawa Hospital Research Institute, Ottawa, ON K1H 8L6 Canada; 4 Department of Anesthesiology and Pain Medicine, The Ottawa Hospital, Ottawa, ON K1H 8L6 Canada

**Keywords:** Predatory publishing, Predatory journals, Scholarly communication, Systematic review

## Abstract

**Background:**

The increase in the number of predatory journals puts scholarly communication at risk. In order to guard against publication in predatory journals, authors may use checklists to help detect predatory journals. We believe there are a large number of such checklists yet it is uncertain whether these checklists contain similar content. We conducted a systematic review to identify checklists that help to detect potential predatory journals and examined and compared their content and measurement properties.

**Methods:**

We searched MEDLINE, Embase, PsycINFO, ERIC, Web of Science and Library, and Information Science & Technology Abstracts (January 2012 to November 2018); university library websites (January 2019); and YouTube (January 2019). We identified sources with original checklists used to detect potential predatory journals published in English, French or Portuguese. Checklists were defined as having instructions in point form, bullet form, tabular format or listed items. We excluded checklists or guidance on recognizing “legitimate” or “trustworthy” journals. To assess risk of bias, we adapted five questions from *A Checklist for Checklists* tool a priori as no formal assessment tool exists for the type of review conducted.

**Results:**

Of 1528 records screened, 93 met our inclusion criteria. The majority of included checklists to identify predatory journals were in English (*n* = 90, 97%), could be completed in fewer than five minutes (*n* = 68, 73%), included a mean of 11 items (range = 3 to 64) which were not weighted (*n* = 91, 98%), did not include qualitative guidance (*n* = 78, 84%), or quantitative guidance (n = 91, 98%), were not evidence-based (n = 90, 97%) and covered a mean of four of six thematic categories. Only three met our criteria for being evidence-based, i.e. scored three or more “yes” answers (low risk of bias) on the risk of bias tool.

**Conclusion:**

There is a plethora of published checklists that may overwhelm authors looking to efficiently guard against publishing in predatory journals. The continued development of such checklists may be confusing and of limited benefit. The similarity in checklists could lead to the creation of one evidence-based tool serving authors from all disciplines.

## Background

The influx of predatory publishing along with the substantial increase in the number of predatory journals pose a risk to scholarly communication [1, 2]. Predatory journals often lack an appropriate peer-review process and frequently are not indexed [3], yet authors are required to pay an article processing charge. The lack of quality control, the inability to effectively disseminate research and the lack of transparency compromise the trustworthiness of articles published in these journals. Until recently, no agreed-upon definition of predatory journals existed. However, through a consensus process [4], an international group of researchers, journal editors, funders, policy makers, representatives of academic institutions, and patient partners, developed a definition of predatory journals and publishers. The group recognized that identifying predatory journals and publishers was nuanced; not all predatory journals meet all ‘predatory criteria’ nor do they meet each criterion at the same level. Thus, in defining predatory journals and publishers, the group identified four main characteristics that could characterize journals or publishers as predatory: “Predatory journals and publishers are entities that prioritize self-interest at the expense of scholarship and are characterized by false or misleading information, deviation from best editorial/publication practices, lack of transparency, and/or use of aggressive and indiscriminate solicitation practices.” [4]. Lists of suspected predatory journals and publishers are also available, although different criteria for inclusion are used [5].

Various groups have developed checklists to help prospective authors and/or editors identify potential predatory journals; these are different from efforts, such as “Think. Check. Submit.” to identify legitimate journals. Anecdotally, we have recently noticed a steep rise in the number of checklists developed specifically to identify predatory journals, although to our knowledge this has not been quantified previously. Further, we are unaware of any research looking at the uptake of these checklists. On the one hand, the development of these checklists – practical tools to help detect potential predatory journals – may lead to a substantial decrease in submissions to these journals. On the other hand, large numbers of checklists with varying content may confuse authors, and possibly make it more difficult for them to choose any one checklist, if any at all, as suggested by the *choice overload* hypothesis [6]. That is, the abundance of conflicting information could result in users not consulting any checklists. Additionally, the discrepancies between checklists could impact the credibility of each one. Thus, these efforts to reduce the number of submissions to predatory journals will be lost. Therefore, we performed a systematic review of peer reviewed and gray literature that include checklists to help detect potential predatory journals in order to identify the number of published checklists and to examine and compare their content and measurement properties.

## Methods

We followed standard procedures for systematic reviews and reported results according to Preferred Reporting Items for Systematic reviews and Meta-Analyses (PRISMA) guidelines [7]. The project protocol was publicly posted prior to data extraction on the Open Science Framework (http://osf.io/g57tf).

### Data sources and searches

An experienced medical information specialist (BS) developed and tested the search strategy using an iterative process in consultation with the review team. The strategy was peer reviewed by another senior information specialist prior to execution using the Peer Review of Electronic Search Strategies (PRESS) Checklist [8] (see Additional file 1).

We searched multiple databases with no language restrictions. Using the OVID platform, we searched Ovid MEDLINE® ALL (including in-process and epub-ahead-of-print records), Embase Classic + Embase, PsycINFO and ERIC. We also searched Web of Science and the Library, Information Science and Technology Abstracts (LISTA) database (Ebsco platform). The LISTA search was performed on November 16, 2018 and the Ovid and Web of Science searches were performed on November 19, 2018. Retrieval was limited to the publication dates 2012 to the present. We used 2012 as a cut-off since data about predatory journals were first collected in 2010, [9] and became part of public discourse in 2012 [10]. The search strategy for the Ovid databases is included in Additional file 2.

In order to be extensive in our search for checklists that identify potential predatory journals, we identified and then searched two relevant sources of gray literature, based on our shared experiences in this field of research: university library websites and YouTube. Neither search was restricted by language. We used the Shanghai Academic Rankings of World Universities (http://www.shanghairanking.com/ARWU-Statistics-2018.html) to identify university library websites of the top 10 universities in each of the four world regions (Americas, Europe, Asia / Oceania, Africa). We chose this website because it easily split the world into four regions and we saw this as an equitable way to identify institutions and their libraries. As our author group is based in Canada, we wanted to highlight the universities in our region and therefore identified the library websites of Canada’s most research-intensive universities (U15) (search date January 18, 2019) and searched their library websites. We also searched YouTube for videos that contained checklists (search date January 6, 2019). We limited our YouTube search to the top 50 results filtered by “relevance” and used a private browser window. Detailed methods of these searches are available on the Open Science Framework (http://osf.io/g57tf).

### Eligibility criteria

#### Inclusion criteria

Our search for studies was not restricted by language, however, for reasons of feasibility, we included studies and/or original checklists developed or published in English, French or Portuguese (languages spoken by our research team). We defined checklist as a tool whose purpose is to detect a potential predatory journal and the instructions are in point form / bullet form / tabular format / listed items. To qualify as an original checklist, the items had to have been identified and/or developed by the study authors or include a novel combination of items from multiple sources, or an adaptation of another checklist plus items added by the study authors. We included studies that discussed the development of an original checklist. When a study referenced a checklist, but did not describe the development of the checklist, we searched for the paper that discussed the development of the original checklist and included that paper.

#### Exclusion criteria

Checklists were not considered original if items were hand-picked from one other source; for example, if authors identified the five most salient points from an already existing checklist.

We did not include lists or guidance on recognizing a “legitimate” or “trustworthy” journal. We stipulated this exclusion criterion since our focus was on tools that specifically identify predatory journals, not tools that help to recognize legitimate journals.

### Study selection

**Fig. 1:**
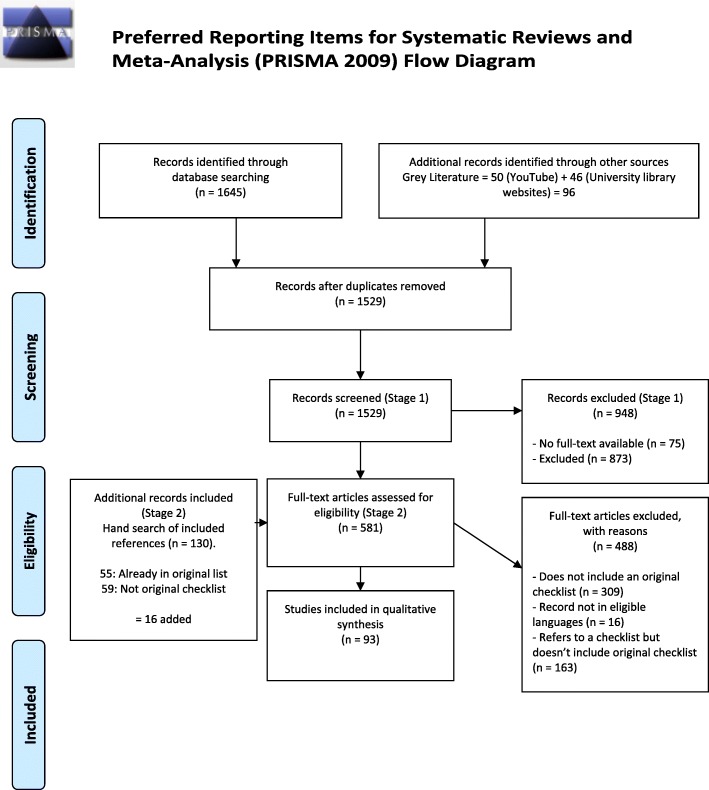
PRISMA flow diagram

Following de-duplication of the identified titles, we screened records using the online systematic review software program Distiller Systematic Review (DSR) (Evidence Partners Inc., Ottawa, Canada). For each stage of screening, data extraction and risk of bias assessment, we pilot tested a 10% sample of records among five to six reviewers. Screening was performed in two stages: Stage 1: title and abstract; Stage 2: full-text screening (see Fig. 1). Both stages were completed by two reviewers independently and in duplicate. At both stages, discrepancies were resolved either through consensus or third party adjudication.

### Data extraction and risk of Bias assessment

For each eligible study, two reviewers independently extracted relevant data into DSR and a third reviewer resolved any conflicts. The extracted data items were as follows: 1- checklist name, 2- number of items in the checklist, 3- whether the items were weighted, 4- the number of thematic categories covered by the checklist (six-item list developed by Cobey et al. [3]), 5- publication details (name of publication, author and date of publication), 6- approximate time to complete checklist (reviewers used a timer to emulate the process that a user would go through to use the checklist and recorded the time as 0–5 min, 6–10 min, or more than 10 min), 7- language of the checklist, 8- whether the checklist was translated and into what language(s), 9- methods used to develop the checklist (details on data collection, if any), 10- whether there was qualitative guidance (instructions on how to use the checklist and what to do with the results) and/or 11- quantitative guidance (instructions on summing the results or quantitatively assessing the results to inform a decision). The list of extracted data items can be found on the Open Science Framework (https://osf.io/na756/).

In assessing checklists identified via YouTube, we extracted only data items that were presented visually. Any item or explanation that was delivered by audio only was not included in our assessment. We used the visual presentation of the item to be a sign that the item was formally included in the checklist. For example, if presenters only talked about a checklist item but did not have it on a slide in the video or in a format that could be seen by those watching the video, we did not extract this data.

To assess risk of bias, we developed an a priori list of five questions for the purpose of this review, adapted from *A Checklist for Checklists* tool [11], and principles of internal and external validity [12]. The creation of a novel tool to assess risk of bias was necessary since there is no appropriate formal assessment tool that exists for the type of review we conducted. Our author group looked over the three areas identified in the Checklist for Checklists tool (Development, Drafting and Validation). Based on extensive experience working with reporting guidelines (DM), which are checklists, we chose a feasible number of items from each of the three categories to be used in our novel tool. We pilot tested the items among our author group to ensure that all categories were captured adequately, and that the tool could be used feasibly.

We used the results of this assessment to determine whether the checklist was evidence-based. We assigned each of the five criteria (listed below) a judgment of “yes” (i.e. low risk of bias), “no” (i.e. high risk of bias) or “cannot tell” (i.e. unclear risk of bias) (see coding manual with instructions for assessment to determine risk of bias ratings: https://osf.io/sp4vx/). If the checklist scored three or more “yes” answers on the questions below, assigning the checklist an overall low risk of bias, we considered it evidence-based. We made this determination based on the notion that a low risk of bias indicates that there is a low risk of systematic error across results. Two reviewers independently assessed data quality in DSR and discrepancies were resolved through discussion. A third reviewer was called to resolve any remaining conflicts.

The five criteria, adapted from the Checklist for Checklists tool [11], used to assess risk of bias in this review were as follows:

**Table:** 

1. Did the developers of the checklist represent more than one stakeholder group (e.g. researchers, academic librarians, publishers)? 2. Did the developers report gathering any data for the creation of the checklist (i.e. conduct a study on potential predatory journals, carry out a systematic review, collect anecdotal data)? 3. Does the checklist meet at least one of the following criteria: 1- Has title that reflects its objectives; 2- Fits on one page; 3- Each item on the checklist is one sentence? 4. Was the checklist pilot-tested or trialed with front-line users (e.g. researchers, students, academic librarians)? 5. Did the authors report how many criteria in the checklist a journal must meet in order to be considered predatory?	

In assessing websites, we used a “two-click rule” to locate information. Once on the checklist website, if we did not find the information within two mouse clicks, we concluded no information was available.

### Data synthesis and analysis

We examined the checklists qualitatively and conducted qualitative comparisons of the items. We compared the items in the included checklists to gauge their agreement on content by item and overall. We summarized the checklists in table format to facilitate inspection and discussion of findings. Frequencies and percentages were used to present characteristics of the checklists. We used the list developed by Shamseer et al. [13] as the reference checklist and compared our results to this list. We chose this as the reference list because of the rigorous empirical data generated by authors to ascertain characteristics of potential predatory journals.

## Results

### Deviations from our protocol

We refined our definition of an *original* checklist to exclude checklists that were comprised of items taken solely from another checklist. Checklists made up of items taken from more than one source were considered original even when the developers did not create the checklist items themselves. For reasons of feasibility, we did not search the reference lists in these checklists to identify further potentially relevant studies.

To screen the titles and abstracts, we had anticipated using the liberal accelerated method where only one reviewer is required to include citations for further assessment at full-text screening and two reviewers are needed to exclude a citation [14]. Instead, we used the traditional screening approach: we had two reviewers screen records independently and in duplicate. We changed our screening methods because it became feasible to use the traditional screening approach, which also reduced the required number of full-text articles to be ordered.

After completing data collection, we recognized that checklists were being published in discipline-specific journals, within biomedicine. We wanted to determine what disciplines were represented and in what proportion. We conducted a scan of the journals and used an evolving list of disciplines to assign to the list of journals, i.e. we added disciplines to the evolving list as we came across them.

### Study selection

Following the screening of 1529 records, we identified 93 original checklists to be included in our study (see full details in Fig. 1).

### Checklist characteristics

We identified 53 checklists identified through our search of electronic databases. The numbers of checklists identified increased over time: one each in 2012 [10], 2013 [15], rising to 16 in 2017 [13, 16–30] and 12 in 2018 [31–42]. We identified 30 original checklists [1, 43–71] from university library websites. More checklists were published in more recent years (2017 = 4 [45–48]; 2018 = 7 [49–55]; 2019 = 11 [56–66]; five checklists listed no publication date). We identified 10 more checklists from YouTube [72–81] that included one uploaded in 2015 [72], six in 2017 [73–78] and three in 2018 [79–81]. See Table 1 for full checklist characteristics.

**Table 1:** Characteristics of checklists (oldest to most recently published)

Study	Checklist name	Number of items	Items weighted Y/N	Time to complete min	Methods used to develop checklist (NR = Not reported)	Qualitative guidance Y/N	Quantitative guidance Y/N
***Checklists from electronic journal databases n = 53***							
[REFERENCE CHECKLIST] Shamseer, 2017 [13]	Salient characteristics of potential predatory journals	13	N	0–5	Cross-sectional analysis 93 predatory journals, 99 OA, 100 subscription based journals assessed	No	No
Beall, 2012 [10]	Criteria for Determining Predatory Open-Access Publishers	5	No	10+ min	Criteria based on AOSPA^a^ Code of Conduct, two COPE^b^ publications	No	No
Beall, 2013 [15]	Some warning signs of questionable publishers	7	No	0–5 min	Observational research on own emails received	No	No
Crawford, 2014 [82]	No title	11	No	6–10 min	Assessed all criteria in Beall’s criteria	Yes	No
Knoll, 2014 [83]	Avoiding Predatory OA Journals	17	No	0–5 min	Works cited	No	No
Lukic, 2014 [84]	No title	13	No	0–5 min	Multiple references	No	No
Beall, 2015 [85]	Criteria for Determining Predatory Open-Access Publishers	5	No	10+ min	Criteria based on COPE documents: Code of Conduct and Principles of Transparency and Best Practices in Scholarly Publication	No	No
Bhad, 2015 [86]	How should one suspect a journal could be a predatory journal?	9	No	0–5 min	NR	No	No
Bradley-Springer, 2015 [87]	No title	6	No	0–5 min	Multiple references	No	No
Hemmat Esfe, 2015 [88]	Features of the Fake Journals	9	No	0–5 min	NR	No	No
INANE Predatory Publishing Practices Collaborative, 2015 [89]	Guidelines for evaluating the integrity of a journal / Red flags	7	No	0–5 min	Limited literature review	No	No
Pamukcu Gunaydin, 2015 [90]	No title	10	No	0–5 min	Authors’ top 10 based on other references	No	No
Proehl, 2015 [91]	Guidelines for evaluating the integrity of a journal - Red flags	7	No	0–5 min	References to other checklists: COPE etc.	No	No
Stone, 2015 [92]	Guidelines for evaluating the integrity of a journal	6	No	0–5 min	Other credible resources: COPE, INANE^c^, other	No	No
Yucha, 2015 [93]	Guidelines for Evaluating the Integrity of a Journal - Red flags	10	No	0–5 min	Multiple references to other checklists	No	No
Cariappa, 2016 [94]	Telltale signs - Something is wrong!	7	No	0–5	Some literature cited	No	No
Carroll, 2016 [95]	Common Practices of Predatory Open Access Publications	4	No	0–5 min	Limited literature review	No	No
Dadkhah, 2016 [96]	Criteria to rank predatory journals	14	Yes	10+ min	Observational study of 150 journals 80 predatory, 70 non	Yes	Yes
Fraser, 2016 [97]	Red Flags for Recognizing Predatory Journals	6	No	0–5 min	Two citations	No	No
Glick, 2016 [98]	What you can expect from a predatory publisher	7	No	0–5 min	Multiple citations	No	No
Glick, 2016a [99]	Clues suggesting a “predatory” journal	11	No	0–5 min	Multiple references	No	No
Hansoti, 2016 [100]	Overall Approach to Choosing the Journal	11	No	0–5 min	Extensive literature review	Yes	No
Morley, 2016 [101]	10 steps to spot a predatory publisher	10	No	0–5 min	A few citations	Yes	No
Nolan, 2016 [102]	None section title exists but not title	5	No	0–5 min	None noted	No	No
Ward, 2016 [103]	No title	8	No	6–10 min	None listed	No	No
Abadi, 2017 [16]	No title	26	No	6–10 min	NR	No	No
Balehegn, 2017 [30]	No title	5	No	0–5 min	References	No	No
Berger, 2017 [17]	Detailed Characteristics of Predatory Journals	15	No	6–10 min	NR	No	No
Das, 2017 [18]	How to identify predators?	15	No	0–5 min	Two citations	No	No
Erfanmanesh 2017 [19]	No title	18	No	6–10 min	Multiple references	No	No
Eriksson, 2017 [20]	Characteristics of a predatory journal	25	No	10+	Limited literature review	No	No
Janodia, 2017 [21]	No title	9	No	6–10 min	NR	Yes	No
Khan, 2017 [22]	Attributes, characteristics and practices of potential predatory journals	9	No	0–5 min	Citations	No	No
Klyce, 2017 [23]	Common characteristics of predatory journals	13	No	0–5 min	Limited literature review	No	No
Manca, 2017 [24]	No title	6	No	0–5 min	Limited literature review	No	No
Miller, 2017 [25]	Signs of a Predatory Publisher	8	No	0–5 min	NR	No	No
Misra, 2017 [26]	Red flags based on which one may suspect the legitimacy of a journal	17	No	0–5 min	Literature review and authors’ experiences	No	No
Mouton, 2017 [27]	Comparing the characteristics of good practice in scholarly publishing with those of predatory publishing	7	No	0–5 min	In-depth assessment of journals identified by Beall’s list where South African authors published	No	No
Oren, 2017 [28]	Obvious signs of predatory journals	7	No	0–5 min	NR	No	No
Shamseer, 2017 [13]	Salient characteristics of potential predatory journals	13	No	0–5 min	Observational study 93 predatory journals, 99 OA^d^, 100 subscription-based journals assessed	No	No
Stratton, 2017 [29]	Characteristics of Health and Medical Journal Publishing Formats - Open Access Predatory	4	No	0–5 min	Cited references	No	No
Ajuwon, 2018 [33]	Characteristics of Predatory Publishers and Journals	12	No	0–5 min	Citations from other sources	No	No
Bowman, 2018 [34]	Identifying Predatory Journals and Publishers	29	No	6–10 min	NR	No	No
Gerberi, 2018 [35]	Quick List of Predatory Publisher Warning Signs	7	No	0–5 min	Limited literature review	No	No
Kokol, 2018 [37]	No title	4	No	0–5	Analysis of papers 2013–2017 predatory Beall’s vs non	No	No
Lewinski, 2018 [38]	Eight tips to identify a predatory journal or publisher	8	No	0–5	NR	Yes	No
McCann, 2018 [31]	Guidelines for authors to avoid predatory publishers	25	No	0–5 min	Brief literature review	Yes	No
Memon, 2018 [39]	No title	14	No	6–10 min	Collecting emails and web pages of each journal /publisher. Used Beall’s list, PubMed, DOAJ^e^, Thomson and Reuters now Clarivate Analytics	No	No
Nnaji, 2018 [40]	No title	11	No	6–10 min	Two references	No	No
O’Donnell, 2018 [32]	Identifying a predator	17	No	6–10 min	Other evidence-based checklist	No	No
Pamukcu Gunaydin, 2018 [36]	How to avoid sending your work to a predatory journal	5	No	0–5	Limited literature review	No	No
Power, 2018 [41]	No title	11	No	6–10 min	References COPE, INANE	No	No
Richtig, 2018 [42]	Criteria identified or suggested in the literature that can potentially be used to identify predatory journals	13	No	6–10	Literature review	No	No
Wikipedia, 2019 [104]	No title	10	No	0–5 min	Multiple citations	No	No
***Checklists from university library websites n = 30***							
[REFERENCE CHECKLIST] Shamseer, 2017 [13]	Salient characteristics of potential predatory journals	13	N	0–5	Cross sectional analysis 93 predatory journals, 99 OA, 100 subscription-based journals assessed	No	No
Carlson, 2014 [43]	None	11	No	0–5 min	Based on personal experiences looking into questionable OA journals	No	No
Clark, 2015 [1]	None	5	No	0–5 min	NR	No	No
University of Edinburgh, 2015 [44]	Some warning signs to look out for	4	No	0–5 min	NR	No	No
Africa Check, 2017 [45]	None	7	No	0–5 min	NR	No	No
Cabell’s – Clarivate, 2017 [46]	None	64	No	10+ min	Analysis by specialists see: https://www2.cabells.com/about-blacklist	No	No
Duke University Medical Center, 2017 [47]	Be iNFORMEd Checklist	6	No	0–5 min	NR	Yes	No
University of Calgary, 2017 [48]	No title	6	No	0–5 min	NR	No	No
Coopérer en information scientifique et technique, 2018 [49]	No title	35	No	10+ min	NR	No	No
Eaton, University of Calgary 2018 [50]	No title	12	No	0–5 min	Other sources cited	Yes	No
Lapinksi, Harvard, 2018 [51]	No title	3	No	0–5 min	NR	Yes	No
Sorbonne Université, 2018 [52]	Comment repérer un éditeur prédateur	12	No	0–5 min	NR	No	No
University of Alberta, 2018 [54]	No title	19	No	6–10 min	NR	No	No
University of British Columbia, 2018 [53]	No title	16	No	6–10 min	NR	No	No
University of Toronto Libraries, 2018 [55]	Identifying Deceptive Publishers: A Checklist	22	Yes	6–10 min	NR	Yes	Yes
Dalhousie University, 2019 [56]	How to recognize predatory journals	6	No	0–5 min	NR	No	No
McGill University, 2019 [57]	No title	4	No	0–5 min	NR	No	No
McMaster University, 2019 [58]	No title	6	No	0–5 min	NR	No	No
Prater, American Journal Experts, 2019 [59]	No title	8	No	0–5 min	NR	Yes	No
Ryerson University Library, 2019 [60]	No title	5	No	0–5 min	NR	No	No
Université Laval, 2019 [61]	No title	8	No	0–5 min	NR	No	No
University of Cambridge, 2019 [62]	No title	9	No	6–10 min	NR	No	No
University of Pretoria, 2019 [63]	No title	19	No	0–5 min	NR	No	No
University of Queensland Library, 2019 [65]	No title	6	No	0–5 min	NR	No	No
University of Queensland Library, 2019a [64]	Red Flags for Open Access Journals	9	No	0–5 min	NR	Yes	No
University of Witwatersrand, 2019 [66]	Predatory Publisher Checklist	26	No	10+ min	NR	No	No
Canadian Association of Research Libraries, ND [67]	How to assess a journal A.K.A. How not to publish in an undesirable journal	12	No	0–5 min	NR	Yes	No
Columbia University Libraries, ND [68]	No title	5	No	0–5 min	NR	Yes	No
Technion Library, ND [69]	No title	10	No	0–5 min	NR	No	No
UC Berkley, ND [70]	No title	3	No	0–5 min	Cited 1 paper	No	No
University of Ottawa Scholarly Communication, ND [71]	No title	12	No	0–5 min	NR	No	No
***Checklists from YouTube n = 10***							
[REFERENCE CHECKLIST] Shamseer, 2017 [13]	Salient characteristics of potential predatory journals	13	N	0–5	Cross sectional analysis 93 predatory journals, 99 OA, 100 subscription-based journals assessed	No	No
Robbins, Western Sydney University, 2015 [72]	Red Flags	9	No	6–10 min	NR	No	No
Attia, 2017 [73]	Spot Predatory Publishers	4	No	0–5 min	NR	No	No
Kysh, USC Keck School of Medicine, 2017 [74]	Characteristics of Predatory Publishers	9	No	0–5 min	NR	No	No
McKenna, Rhodes University, 2017 [75]	Predatory Publications: Shark Spotting	7	No	0–5 min	NR	No	No
Nicholson, University of Witwatersrand, 2017 [76]	Cautionary Checklist	36	No	10+ min	NR	No	No
Raszewski, 2017 [77]	What to watch out for	4	No	0–5 min	NR	No	No
Seal-Roberts, Springer Healthcare, 2017 [78]	So how do we recognize a predatory publisher?	10	No	0–5 min	NR	No	No
Menon, SCMS Group of Educational Institutions, India and Berryman, Cabell’s, 2018 [79]	Characteristics of Predatory Journals	7	No	0–5 min	NR	No	No
Office of Scholarly Communication, Cambridge University, 2018 [80]	None	12	No	0–5 min	NR	No	No
Weigand, UNC Libraries, 2018 [81]	No title	5	No	0–5 min	NR	No	No

^a^*OASPA* Open Access Scholarly Publishers Association

^b^*COPE* Committee on Publication Ethics

^c^*INANE* International Academy of Nursing Editors

^d^*OA* Open Access

^e^*DOAJ* Directory of Open Access Journals

#### Language and translation

Almost all checklists were published in English (*n* = 90, 97%), and the remaining checklists in French (*n* = 3, 3%) [49, 52, 61]. Two additional English checklists identified through university library websites were translated into French [55, 67] and one was translated into Hebrew [69].

#### Approximate time for user to complete checklist, number of items per checklist and weighting

Most checklists could be completed within five minutes (*n* = 68, 73%); 17 checklists (18%) could be completed in six to 10 min [16, 17, 19, 21, 32, 34, 39–42, 53–55, 62, 72, 82, 103] and eight checklists (9%) took more than 10 min to complete [10, 20, 46, 49, 66, 76, 85, 96]. Checklists contained a mean of 11 items each, and a range of between three and 64 items. Items were weighted in two checklists [55, 96].

#### Qualitative and quantitative guidance

Qualitative guidance on how to use the results of checklists was provided on 15 checklists (16%) [21, 31, 38, 47, 50, 51, 55, 59, 65, 67, 68, 82, 96, 100, 101], and quantitative guidance was provided on two checklists [55, 96], i.e. prescribing a set number of criteria that would identify the journal or publisher as predatory.

#### Methods used to develop checklists

In order to develop the checklists, authors noted using analysis by specialists [46], information from already existing checklists [85, 91, 93], using existing literature on predatory journals to pick the most salient features to create a new checklist [31, 42, 100], developing checklists after empirical study [13, 27, 39, 96] or after personal experiences [15].

### Risk of bias assessment

Among all 93 checklists, there were three (3%) assessed as evidence-based [27, 96, 100] (see Table 2 for detailed risk of bias assessment results including whether a checklist was determined to be evidence-based, i.e. rated as low risk of bias for at least three of the criteria).

**Table 2:** Risk of bias assessment. Three ‘Yes’ assessments results in an overall assessment of evidence-based

Study	Represent 1+ stakeholder groups (Y/N/U)*	Gather data for checklist development (Y/N/Only citations/ U)	Does the checklist meet at least one of these criteria? (count only the last column in total)				Pilot test (Y/N/U)	Includes number of criteria to be considered predatory (Y/N)	Overall assessment (is it evidence-based?) (Y/N)
			Title (Y/N)	Fits on one page (Y/N)	Each item one sentence (Y/N)	Meets at least one of these (Y/N)			
***Checklists from electronic journal databases (n = 53)***									
[REFERENCE CHECKLIST] Shamseer, 2017 [13]	U	Y	Y	Y	Y	Y	U	N	N
Beall, 2012 [10]	U	U	N	N	N	N	U	N	N
Beall, 2013 [15]	U	Y	Y	Y	Y	Y	U	N	N
Crawford, 2014 [82]	U	U	N	Y	Y	Y	U	N	N
Knoll, 2014 [83]	U	Only citations	Y	Y	N	Y	U	N	N
Lukic, 2014 [84]	U	Only citations	N	Y	N	Y	U	N	N
Beall, 2015 [85]	U	Only citations	N	N	N	N	U	N	N
Bhad, 2015 [86]	U	U	Y	Y	Y	Y	U	N	N
Bradley-Springer, 2015 [87]	U	Only citations	N	Y	N	Y	U	N	N
Hemmat Esfe, 2015 [88]	U	Only citations	N	Y	Y	Y	U	N	N
INANE Predatory Publishing Practices Collaborative, 2015 [89]	U	U	Y	Y	Y	Y	U	N	N
Pamukcu Gunaydin, 2015 [90]	U	Only citations	N	Y	N	Y	U	N	N
Proehl, 2015 [91]	U	U	N	Y	Y	Y	U	N	N
Stone, 2015 [92]	U	Only citations	N	Y	Y	Y	U	N	N
Yucha, 2015 [93]	U	U	Y	Y	Y	Y	U	N	N
Cariappa, 2016 [94]	U	Only citations	Y	Y	Y	Y	U	N	N
Carroll, 2016 [95]	U	Y	Y	Y	Y	Y	U	N	N
Dadkhah, 2016 [96]	U	Y	Y	Y	Y	Y	Y	Y	Y
Fraser, 2015 [97]	U	Only citations	Y	Y	Y	Y	U	N	N
Glick, 2016 [98]	U	U	Y	Y	Y	Y	U	N	N
Glick, 2016a [99]	U	Only citations	Y	Y	Y	Y	U	N	N
Hansoti, 2016 [100]	Y	Y	N	Y	N	Y	U	N	Y
Morley, 2016 [101]	U	U	N	N	N	N	U	N	N
Nolan, 2016 [102]	U	U	N	Y	N	Y	U	N	N
Ward, 2016 [103]	U	U	N	N	N	N	U	N	N
Abadi, 2017 [16]	U	U	N	Y	Y	Y	U	N	N
Balehegn, 2017 [30]	U	Only citations	N	Y	Y	Y	U	N	N
Berger, 2017 [17]	U	U	N	Y	N	Y	U	N	N
Das, 2017 [18]	U	Only citations	Y	Y	Y	Y	U	N	N
Erfanmanesh, 2017 [19]	U	Only citations	Y	Y	Y	Y	U	N	N
Eriksson, 2017 [20]	U	U	Y	Y	Y	Y	U	N	N
Janodia, 2017 [21]	U	U	Y	Y	N	Y	U	N	N
Khan, 2017 [22]	Y	Only citations	Y	Y	N	Y	U	N	N
Klyce, 2017 [23]	U	Only citations	Y	Y	Y	Y	U	N	N
Manca, 2017 [24]	U	Only citations	N	Y	N	Y	U	N	N
Miller, 2017 [25]	U	U	Y	Y	Y	Y	U	N	N
Misra, 2017 [26]	U	Y	Y	Y	Y	Y	U	N	N
Mouton, 2017 [27]	U	U	Y	Y	N	Y	Y	Y	Y
Oren, 2017 [28]	U	U	Y	Y	Y	Y	U	N	N
Shamseer, 2017 [13]	U	Y	Y	Y	Y	Y	U	N	N
Stratton, 2017 [29]	U	U	Y	Y	Y	Y	U	N	N
Ajuwon, 2018 [33]	U	Only citations	Y	Y	Y	Y	U	N	N
Bowman, 2018 [34]	U	U	Y	Y	N	Y	U	N	N
Gerberi, 2018 [35]	U	Only citations	Y	Y	N	Y	U	N	N
Pamukcu Gunaydin, 2018 [36]	U	Only citations	Y	Y	N	Y	U	N	N
Kokol, 2018 [37]	U	Y	N	Y	Y	Y	U	N	N
Lewinski, 2018 [38]	U	Y	Y	Y	N	Y	U	N	N
McCann, 2018 [31]	U	Y	Y	Y	Y	Y	U	N	N
Memon, 2018 [39]	U	Y	Y	Y	Y	Y	U	N	N
Nnaji, 2018 [40]	U	Only citations	Y	N	N	Y	U	N	N
O’Donnell, 2018 [32]	U	Only citations	N	N	N	N	U	N	N
Power, 2018 [41]	U	Only citations	N	N	N	N	U	N	N
Richtig, 2018 [42]	U	Only citations	Y	Y	N	Y	U	N	N
Wikipedia, 2019 [104]	U	Only citations	N	Y	Y	Y	U	N	N
***Checklists from university library websites (n = 30)***									
[REFERENCE CHECKLIST] Shamseer, 2017 [13]	U	Y	Y	Y	Y	Y	U	N	N
Carlson, 2014 [43]	U	Y	N	N	N	N	U	N	N
Clark, 2015 [1]	U	U	N	Y	N	Y	U	N	N
University of Edinburgh, 2015 [44]	U	U	Y	Y	Y	Y	U	N	N
Africa Check, 2017 [45]	U	Only citations	N	Y	N	Y	U	N	N
Cabell’s - Clarivate, 2017 [46]	Y	U	N	N	Y	Y	U	N	N
Duke University Medical Center, 2017 [47]	U	U	N	N	N	N	U	N	N
University of Calgary, 2017 [48]	U	U	N	Y	N	Y	U	N	N
Coopérer en information scientifique et technique, 2018 [49]	U	U	N	N	Y	Y	U	N	N
Eaton, University of Calgary, 2018 [50]	U	Y	N	Y	Y	Y	U	N	N
Lapinski, Harvard University, 2018 [51]	U	U	N	N	N	N	U	N	N
Sorbonne Université, 2018 [52]	U	U	Y	Y	Y	Y	U	N	N
University of Alberta, 2018 [54]	U	U	N	N	N	N	U	N	N
University of British Columbia, 2018 [53]	U	Only citations	Y	N	N	Y	U	N	N
University of Toronto Libraries, 2018 [55]	Y	U	N	N	N	N	U	Y	N
Dalhousie University, 2019 [56]	U	U	Y	Y	N	Y	U	N	N
McGill University, 2019 [57]	U	U	N	Y	N	Y	U	N	N
McMaster University, 2019 [58]	U	U	N	Y	Y	Y	U	N	N
Prater - American Journal Experts, 2019 [59]	Y	U	N	N	N	N	U	N	N
Ryerson University Library, 2019 [60]	U	U	N	Y	N	Y	U	N	N
Université Laval, 2019 [61]	U	U	N	Y	N	Y	U	N	N
University of Cambridge, 2019 [62]	U	U	N	Y	N	Y	U	N	N
University of Pretoria, 2019 [63]	U	U	N	Y	Y	Y	U	N	N
University of Queensland Library, 2019 [65]	U	U	N	Y	N	Y	U	N	N
University of Queensland Library, 2019a [64]	U	U	N	Y	N	Y	U	N	N
University of Witwatersrand, 2019 [66]	U	U	Y	N	Y	Y	U	N	N
Canadian Association of Research Libraries, ND [67]	U	U	N	Y	Y	Y	U	N	N
Columbia University Libraries, ND [68]	U	U	N	Y	N	Y	U	N	N
Technion Library, ND [69]	U	U	N	Y	N	Y	U	N	N
UC Berkeley, ND [70]	U	U	N	Y	N	Y	U	N	N
University of Ottawa Scholarly Communication, ND [71]	U	U	N	Y	N	Y	U	N	N
***Checklists from YouTube (n = 10) [URLs available at*****https://osf.io/eds9f/*****]***									
[REFERENCE CHECKLIST] Shamseer, 2017 [13]	U	Y (cross-sectional study on journals)	Y	Y	Y	Y	U	N	N
Robbins S, Western Sydney University, 2015 [72]	U	U	Y	Y	Y	Y	U	N	N
Attia, 2017 [73]	U	U	Y	N	N	Y	U	N	N
Kysh, USC Keck School of Medicine, 2017 [74]	U	U	Y	Y	Y	Y	U	N	N
McKenna, Rhodes University, 2017 [75]	U	U	Y	Y	Y	Y	U	N	N
Nicholson, University of Witwatersrand, 2017 [76]	U	U	Y	N	N	Y	U	N	N
Raszewski, 2017 [77]	U	U	Y	Y	N	Y	U	N	N
Seal-Roberts, Springer Healthcare, 2017 [78]	U	U	Y	Y	Y	Y	U	N	N
Menon, SCMS Group of Educational Institutions, India and Berryman, Cabells, 2018 [79]	U	U	Y	Y	Y	Y	U	N	N
Office of Scholarly Communication, Cambridge University, 2018 [80]	U	U	N	N	Y	Y	U	N	N
Weigand, UNC Libraries, 2018 [81]	U	U	N	Y	Y	Y	U	N	N

*Y Yes, *N* No, *U* Unclear

#### Results for risk of bias criteria

*Criterion #1: representation of more than one stakeholder group in checklist development*

For the majority of checklists (*n* = 88, 94%), it was unclear whether there was representation of more than one stakeholder group in the checklist development process (unclear risk of bias). The remaining five checklists reported the inclusion of more than one stakeholder group (low risk of bias) [22, 46, 55, 59, 100].

*Criterion #2: authors reported gathering data to inform checklist development*

In most studies (*n* = 55, 59%) there was no mention of data gathering for checklist development (unclear risk of bias); in 26 cases (28%), one or two citations were noted next to checklist items, with no other explanation of item development or relevance (high risk of bias) [18, 19, 22–24, 30, 32, 33, 35, 36, 40–42, 45, 53, 83–85, 87, 88, 90, 92, 94, 97, 99, 104]. Twelve records (13%) included a description of authors gathering data to develop a checklist for this criterion (low risk of bias) [13, 15, 26, 31, 37–39, 43, 50, 95, 96, 100].

*Criterion #3: at least one of the following: title that reflected checklist objective; checklist fits on one page; items were one sentence long*

Most checklists were assessed as low risk of bias on this criterion, with 81 of the checklists (87%) meeting at least one of the noted criteria (relevant title, fits on one page, items one sentence long).

*Criterion #4: authors reported pilot testing the checklist*

In the majority of studies (*n* = 91, 98%), authors did not report pilot testing during the checklist development stages (unclear risk of bias).

*Criterion #5: checklist instructions included a threshold number of criteria to be met in order to be considered predatory*

The majority of studies (*n* = 90, 97%), did not include a threshold number of criteria to be met in order for the journal or publisher to be considered predatory (high risk of bias).

### Assessment of the thematic content of the included checklists

We found checklists covered the six thematic categories, as identified by Cobey et al., [3] as follows (see Table 3 for thematic categories and descriptions of categories):

**Table 3:** Thematic categories covered by the checklists (oldest to most recently published)

Study	Categories covered by checklist*					
	Journal Operations	Article	Editorial and Peer Review	Communications	Article Processing Charge	Dissemination, indexing + archiving
***Checklists from electronic journal databases n = 53***						
[REFERENCE CHECKLIST] Shamseer, 2017 [13]	X	X	X	X	X	X
Beall, 2012 [10]	X	X	X	X	X	X
Beall, 2013 [15]	X	X	X	X		
Crawford, 2014 [82]		X	X		X	
Knoll, 2014 [83]	X		X	X	X	X
Lukic, 2014 [84]	X		X	X		X
Beall, 2015 [85]	X	X	X	X	X	X
Bhad, 2015 [86]	X			X	X	X
Bradley-Springer, 2015 [87]	X			X		
Hemmat Esfe, 2015 [88]	X		X	X	X	
INANE Predatory Publishing Practices Collaborative, 2015 [89]	X		X	X		
Pamukcu Gunaydin, 2015 [90]	X	X		X	X	
Proehl, 2015 [91]	X		X	X		
Stone, 2015 [92]	X		X	X		
Yucha, 2015 [93]	X		X	X		
Cariappa, 2016 [94]	X	X	X	X		
Carroll, 2016 [95]	X		X	X		
Dadkhah, 2016 [96]	X		X	X	X	X
Fraser, 2016 [97]	X		X	X	X	
Glick, 2016 [98]	X		X	X	X	
Glick, 2016a [99]		X	X	X	X	
Hansoti, 2016 [100]	X	X	X			X
Morley, 2016 [101]	X			X	X	X
Nolan, 2016 [102]	X		X		X	X
Ward, 2016 [103]	X	X	X	X	X	X
Abadi, 2017 [16]	X	X	X	X	X	X
Balehegn, 2017 [30]						X
Berger, 2017 [17]	X		X	X	X	X
Das, 2017 [18]	X		X		X	X
Erfanmanesh, 2017 [19]	X	X	X	X	X	X
Eriksson, 2017 [20]	X	X	X	X	X	X
Janodia, 2017 [21]	X	X			X	
Khan, 2017 [22]	X		X	X	X	X
Klyce, 2017 [23]	X	X	X	X		X
Manca, 2017 [24]	X	X			X	X
Miller, 2017 [25]	X		X			X
Misra, 2017 [26]	X	X	X	X	X	X
Mouton, 2017 [27]	X		X	X		X
Oren, 2017 [28]	X	X	X	X		
Shamseer, 2017 [13]	X	X	X	X	X	X
Stratton, 2017 [29]			X	X	X	X
Ajuwon, 2018 [33]	X		X	X	X	X
Bowman, 2018 [34]	X	X	X	X	X	X
Gerberi, 2018 [35]	X	X	X			
Kokol, 2018 [37]		X				
Lewinski, 2018 [38]		X	X	X		X
McCann, 2018 [31]	X		X	X	X	X
Memon, 2018 [39]	X		X	X	X	
Nnaji, 2018 [40]	X	X	X	X	X	X
O’Donnell, 2018 [32]	X	X	X	X	X	X
Pamukcu Gunyadin, 2018 [36]	X		X	X		X
Power, 2018 [41]	X		X	X	X	X
Richtig, 2018 [42]	X	X	X	X	X	X
Wikipedia 2019 [104]	X		X		X	X
**TOTALS /53 checklists from electronic journal databases (n, %)**	**47, 89**	**24, 45**	**45, 85**	**42, 79**	**34, 64**	**34, 64**
***Checklists from university library websites n = 30***						
Carlson, 2014 [43]	X			X		
Clark, 2015 [1]	X		X		X	X
University of Edinburgh, 2015 [44]	X			X		
Africa Check, 2017 [45]	X		X			X
Cabell’s – Clarivate, 2017 [46]	X	X	X	X	X	X
Duke University Medical Center, 2017 [47]	X	X	X		X	X
University of Calgary, 2017 [48]	X	X	X		X	
Coopérer en information scientifique et technique, 2018 [49]	X		X	X	X	X
Eaton, University of Calgary, 2018 [50]	X		X	X		X
Lapinksi, Harvard, 2018 [51]						X
Sorbonne Université, 2018 [52]	X		X	X	X	X
University of Alberta, 2018 [54]	X			X	X	X
University of British Columbia, 2018 [53]	X	X	X	X	X	X
University of Toronto Libraries, 2018 [55]	X	X	X	X	X	X
Dalhousie University, 2019 [56]	X			X	X	X
McGill University, 2019 [57]	X		X	X		X
McMaster University, 2019 [58]	X		X	X	X	X
Prater, 2019 [59]	X	X	X		X	
Ryerson University Library, 2019 [60]	X	X	X	X	X	
Université Laval, 2019 [61]	X		X	X	X	X
University of Cambridge, 2019 [62]	X	X	X	X	X	X
University of Pretoria, 2019 [63]	X		X	X	X	X
University of Queensland Library, 2019 [65]	X		X	X	X	X
University of Queensland Library, 2019a [64]	X	X	X			X
University of Witwatersrand, 2019 [66]	X	X	X	X	X	X
Canadian Association of Research Libraries, ND [67]	X	X	X	X	X	X
Columbia University Libraries, ND [68]	X		X			
Technion Library, ND [69]	X	X				X
UC Berkley, ND [70]	X			X	X	X
University of Ottawa Scholarly Communication, ND [71]	X		X	X		X
**Totals /30 checklists from university library websites (n, %)**	**29, 97**	**12, 40**	**23, 77**	**21, 70**	**20, 67**	**24, 80**
***Checklists from YouTube n = 10***						
Robbins S. Western Sydney University, 2015 [72]	X		X	X	X	
Attia, 2017 [73]	X		X	X		
Kysh, USC Keck School of Medicine, 2017 [74]	X		X		X	X
McKenna, Rhodes University, 2017 [75]	X	X		X		
Nicholson, University of Witwatersrand, 2017 [76]	X	X	X	X	X	X
Raszewski 2017 [77]			X	X	X	
Seal-Roberts, Springer Healthcare, 2017 [78]	X		X	X	X	
Menon, SCMS Group of Educational Institutions, India and Berryman, Cabells, 2018 [79]	X		X	X	X	X
Office of Scholarly Communication, Cambridge University, 2018 [80]	X	X	X		X	X
Weigand, 2018 UNC Libraries [81]	X		X	X		
**Totals /10 checklists from YouTube (n, %)**	**9, 90**	**3, 30**	**9, 90**	**8, 80**	**7, 70**	**4, 40**
**TOTAL (n, %)**	**85, 91**	**39, 42**	**77, 83**	**71, 93**	**61, 66**	**62, 67**

*Categories as described by Cobey et al. 2018 [3]

*Journal operations*: 85 checklists (91%) assessed information on the journal’s operations.

*Assessment of previously published articles*: 40 checklists (43%) included questions on the quality of articles published in the journal in question.

*Editorial and peer review process*: 77 checklists (83%) included questions on the editorial and peer review process.

*Communication*: 71 checklists (76%) included an assessment of the manners in which communication is set up between the journal / publisher and the author.

*Article processing charges*: 61 checklists (66%) included an assessment of information on article processing charges.

*Dissemination, indexing and archiving*: 62 checklists (67%) included suggested ways in which submitting authors should check for information on dissemination, indexing and archiving procedures of the journal and publisher.

Across all 93 checklists, a mean of four out of the six thematic categories was covered, demonstrating similar themes covered by all checklists. Twenty percent of checklists (*n* = 19), including the reference checklist, covered all six categories [10, 13, 16, 19, 20, 26, 32, 34, 40, 42, 46, 53, 55, 62, 66, 67, 76, 85, 103]. Assessment of previously published articles was the category least often included in a checklist (*n* = 40, 43%), and a mention of the journal operations was the category most often included in a checklist (*n* = 85, 91%).

### Discipline-specific journals

Of the checklists published in academic journals, 10 (22%) were published in nursing journals [25, 31, 35, 38, 41, 87, 89, 91–93], eight (18%) were published in journals related to general medicine [13, 16, 20, 22, 23, 34, 94, 95], four (9%) in emergency medicine journals [29, 36, 90, 100], four (9%) in information science journals [19, 30, 40, 82], four (9%) in psychiatry and behavioral science journals [18, 24, 83, 86]. The remaining checklists were published in a variety of other discipline-specific journals, within the field of biomedicine, with three or fewer checklists per discipline (e.g. specialty medicine, pediatric medicine, general medicine and surgery, medical education, and dentistry).

## Discussion

Many authors have developed checklists specifically designed to identify predatory journals; the number of checklists developed has increased since 2012, with the majority of checklists published since 2015 (*n* = 81, 87%). Comparing the 93 identified checklists to the reference checklist, we observed that on average, the content of the checklist items were similar, including the categories or domains covered by the checklist; all checklists were also similar on the following: time to complete the checklist, number of items in the checklist (this number does vary considerably, however the average number of items is more consistent with the reference list), and lack of qualitative and quantitative guidance on completing the checklists. Furthermore, only 3% of checklists (*n* = 3) were deemed evidence-based, few checklists weighted any items (*n* = 2, 2%) and few checklists were developed through empirical study (*n* = 4, 4%). Of note, one of the checklists [33] was in a paper in a journal that is potentially predatory.

### Summary of evidence

In total, we identified 93 checklists to help identify predatory journals and/or publishers. A search of electronic databases resulted in 53 original checklists, a search of library websites of top universities resulted in 30 original checklists and a filtered and limited search of YouTube returned 10 original checklists. Overall, checklists could be completed quickly, covered similar categories of topics and were lacking in guidance that would help a user determine if the journal or publisher was indeed predatory.

### Strengths and limitations

We used a rigorous systematic review process to conduct the study. We also searched multiple data sources including published literature, university library websites, globally, and YouTube. We were limited by the languages of checklists we could assess (English, French and Portuguese). However, the majority of academic literature is published in English [105]. Thus, we are confident that we captured the majority of checklists or at least a representative sample. For reasons of feasibility, we were not able to capture all checklists available.

Our reference checklist did not qualify as evidence-based when using our predetermined criteria to assess risk of bias, which could be because the list of characteristics in the reference list was not initially intended as a checklist per se. However, the purpose of the reference checklist was to serve as a reference point for readers, regardless of its qualification as evidence-based or not. Creating a useable checklist tool requires attention not only to the development of the content of items but also to other details, such as pilot testing and making the items succinct, as identified in our risk of bias criteria. This perhaps was not attended to by Shamseer et al. because of the difference in the intended purpose of their list.

Our risk of bias tool was created based on other existing tools and developed through expertise of the authors. Although useful for the purpose of this exercise, the tool remains based on our expert judgment although it does include elements of scientific principles.

We noted that the “Think. Check. Submit.” checklist [106] was referenced in many publications and we believe it is used often as guidance for authors to identify presumed legitimate journals. However, we did not include this checklist in our study because we excluded checklists that help to identify presumed legitimate publications. Instead, our specific focus was on checklists that help to detect potential predatory journals.

## Conclusion

In our search for checklists to help authors identify potential predatory journals, we found great similarity across checklist media and across journal disciplines in which the checklists were published.

Although many of the checklists were published in field-specific journals and / or addressed a specific audience, the content of the lists did not differ much. This could be reflective of the idea that checklist developers are all looking to identify the same items. Only a small proportion of the records included the empirical methods used to develop the checklists, and only a few checklists were deemed evidence-based according to our criteria. We noted that checklists with more items did not necessarily mean that it took longer to complete; this speaks to the level of complexity of some checklists versus others. Importantly, very few authors offered concrete guidance on using the checklists or offered any threshold that would guide authors to identify definitively if the journal was predatory. The lack of checklists providing threshold values could be due to the fact that a definition of predatory journals did not exist until this year [3, 4]. We identify a threshold value as important for the checklist’s usability. Without a recommended or suggested threshold value, checklist users may not feel confident to make a decision on submitting or not submitting to a journal. We are recommending a threshold value as a way for users to actively engage with the checklist and make it a practical tool. The provision of detailed requirements that would qualify a journal as predatory therefore would have been a challenge.

With this large number of checklists in circulation, and the lack of explicit and exacting guidelines to identify predatory publications, are authors at continued risk of publishing in journals that do not follow best publication practices? We see some value in discipline-specific lists for the purpose of more effective dissemination. However, this needs to be balanced against the risk of confusing researchers and overloading them with choice [6]. If most of the domains in the identified checklists are similar across disciplines, would a single list, relevant in all disciplines, result in less confusion and maximize dissemination and enhance implementation?

In our study, we found no checklist to be optimal. Currently, we would caution against any further development of checklists and instead provide the following as guidance to authors:

Look for a checklist that:

1. Provides a threshold value for criteria to assess potential predatory journals, e.g. if the journal contains these *three* checklist items then we recommend avoiding submission;
2. Has been developed using rigorous evidence, i.e. empirical evidence that is described or referenced in the publication.

We note that only one checklist [96] out of the 93 we assessed fulfills the above criteria. There may be other factors (length of time to complete, number of categories covered by the checklist, ease of access, ease of use or other) that may influence usability of the checklist.

Using an evidence-based tool with a clear threshold for identifying potential predatory journals may help reduce the burden of research waste occurring as a result of the proliferation of predatory publications.

## Supplementary information

**Additional file 1.** Peer Review of Electronic Search Strategies (PRESS) Checklist.

**Additional file 2.** Search strategy for the Ovid database.

## Data Availability

All data are available upon request. Supplementary material is available on the Open Science Framework (http://osf.io/g57tf).

## Abbreviations

DSR: Distiller Systematic Review
LISTA: Library, Information Science and Technology Abstracts
PRESS: Peer Review of Electronic Search Strategies
PRISMA: Preferred Reporting Items for Systematic reviews and Meta-Analyses
