# Supplementary material for: Checklists to detect potential predatory biomedical journals: a systematic review
Source: BMC Med. 2020 May 18;18:104. doi: 10.1186/s12916-020-01566-1 (PMC7203891; doi:10.1186/s12916-020-01566-1)
Supplement: Supplementary file 2 — Additional file 2. Search strategy for the Ovid database. [file 12916_2020_1566_MOESM2_ESM.docx]

Ovid Multifile

Database: Embase Classic+Embase <1947 to 2018 November 19>, Ovid MEDLINE(R) ALL <1946 to November 19, 2018>, PsycINFO <1806 to November Week 2 2018>, ERIC <1965 to October 2018>

Search Strategy:

--------------------------------------------------------------------------------

1 (predator* adj3 edit*).tw,kw,kf. (29)

2 (predator* adj3 journal*).tw,kw,kf. (397)

3 (predator* adj3 periodical?).tw,kw,kf. (6)

4 (predator* adj3 publication?).tw,kw,kf. (49)

5 (predator* adj3 publish*).tw,kw,kf. (379)

6 (bogus adj3 edit*).tw,kw,kf. (2)

7 (bogus adj3 journal*).tw,kw,kf. (7)

8 (bogus adj3 periodical?).tw,kw,kf. (0)

9 (bogus adj3 publication?).tw,kw,kf. (0)

10 (bogus adj3 publish*).tw,kw,kf. (1)

11 (dark adj3 edit*).tw,kw,kf. (32)

12 (dark adj3 journal*).tw,kw,kf. (9)

13 (dark adj3 periodical?).tw,kw,kf. (4)

14 (dark adj3 publication?).tw,kw,kf. (2)

15 (dark adj3 publish*).tw,kw,kf. (19)

16 (decepti* adj3 edit*).tw,kw,kf. (21)

17 (decepti* adj3 journal*).tw,kw,kf. (15)

18 (decepti* adj3 periodical?).tw,kw,kf. (0)

19 (decepti* adj3 publication?).tw,kw,kf. (3)

20 (decepti* adj3 publish*).tw,kw,kf. (20)

21 (disreput* adj3 edit*).tw,kw,kf. (0)

22 (disreput* adj3 journal*).tw,kw,kf. (3)

23 (disreput* adj3 periodical?).tw,kw,kf. (0)

24 (disreput* adj3 publication?).tw,kw,kf. (3)

25 (disreput* adj3 publish*).tw,kw,kf. (0)

26 (distrust* adj3 edit*).tw,kw,kf. (1)

27 (distrust* adj3 journal*).tw,kw,kf. (2)

28 (distrust* adj3 periodical?).tw,kw,kf. (0)

29 (distrust* adj3 publication?).tw,kw,kf. (0)

30 (distrust* adj3 publish*).tw,kw,kf. (5)

31 (exploit* adj3 edit*).tw,kw,kf. (107)

32 (exploit* adj3 journal*).tw,kw,kf. (29)

33 (exploit* adj3 periodical?).tw,kw,kf. (1)

34 (exploit* adj3 publication?).tw,kw,kf. (37)

35 (exploit* adj3 publish*).tw,kw,kf. (96)

36 (fake? adj3 edit*).tw,kw,kf. (11)

37 (fake? adj3 journal*).tw,kw,kf. (37)

38 (fake? adj3 periodical?).tw,kw,kf. (0)

39 (fake? adj3 publication?).tw,kw,kf. (4)

40 (fake? adj3 publish*).tw,kw,kf. (20)

41 (hoax$2 adj3 edit*).tw,kw,kf. (1)

42 (hoax$2 adj3 journal*).tw,kw,kf. (5)

43 (hoax$2 adj3 periodical?).tw,kw,kf. (0)

44 (hoax$2 adj3 publication?).tw,kw,kf. (2)

45 (hoax$2 adj3 publish*).tw,kw,kf. (4)

46 (illegitim* adj3 edit*).tw,kw,kf. (3)

47 (illegitim* adj3 journal*).tw,kw,kf. (19)

48 (illegitim* adj3 periodical?).tw,kw,kf. (0)

49 (illegitim* adj3 publication?).tw,kw,kf. (6)

50 (illegitim* adj3 publish*).tw,kw,kf. (12)

51 (mislead* adj3 edit*).tw,kw,kf. (42)

52 (mislead* adj3 journal*).tw,kw,kf. (36)

53 (mislead* adj periodical?).tw,kw,kf. (0)

54 (mislead* adj3 publication?).tw,kw,kf. (57)

55 (mislead* adj publish*).tw,kw,kf. (5)

56 (non-legitim* adj3 edit*).tw,kw,kf. (0)

57 (non-legitim* adj3 journal*).tw,kw,kf. (0)

58 (non-legitim* adj3 periodical?).tw,kw,kf. (0)

59 (non-legitim* adj3 publication?).tw,kw,kf. (0)

60 (non-legitim* adj3 publish*).tw,kw,kf. (0)

61 (questionabl* adj3 edit*).tw,kw,kf. (24)

62 (questionabl* adj3 journal*).tw,kw,kf. (38)

63 (quesionabl* adj3 periodical?).tw,kw,kf. (0)

64 (questionabl* adj3 publication?).tw,kw,kf. (44)

65 (questionabl* adj3 publish*).tw,kw,kf. (48)

66 (racket? adj3 edit*).tw,kw,kf. (0)

67 (racket? adj3 journal*).tw,kw,kf. (1)

68 (racket? adj3 periodical?).tw,kw,kf. (0)

69 (racket? adj3 publication?).tw,kw,kf. (0)

70 (racket? adj3 publish*).tw,kw,kf. (0)

71 (rogue adj3 edit*).tw,kw,kf. (4)

72 (rogue adj3 journal*).tw,kw,kf. (2)

73 (rogue adj3 periodical?).tw,kw,kf. (0)

74 (rogue adj3 publication?).tw,kw,kf. (0)

75 (rogue adj3 publish*).tw,kw,kf. (4)

76 (scam* adj3 edit*).tw,kw,kf. (3)

77 (scam* adj3 journal*).tw,kw,kf. (9)

78 (scam* adj3 periodical?).tw,kw,kf. (0)

79 (scam* adj3 publication?).tw,kw,kf. (0)

80 (scam* adj3 publish*).tw,kw,kf. (6)

81 (sham adj3 edit*).tw,kw,kf. (1)

82 (sham adj3 journal*).tw,kw,kf. (9)

83 (sham adj3 periodical?).tw,kw,kf. (0)

84 (sham adj3 publication?).tw,kw,kf. (1)

85 (sham adj3 publish*).tw,kw,kf. (50)

86 (spam* adj3 edit*).tw,kw,kf. (1)

87 (spam* adj3 journal*).tw,kw,kf. (4)

88 (spam* adj3 periodical?).tw,kw,kf. (0)

89 (spam* adj3 publication?).tw,kw,kf. (2)

90 (spam* adj3 publish*).tw,kw,kf. (5)

91 (unethic* adj3 edit*).tw,kw,kf. (21)

92 (unethic* adj3 journal*).tw,kw,kf. (22)

93 (unethic* adj3 periodical?).tw,kw,kf. (0)

94 (unethic* adj3 publication?).tw,kw,kf. (52)

95 (unethic* adj3 publish*).tw,kw,kf. (51)

96 (unprofessional* adj3 edit*).tw,kw,kf. (1)

97 (unprofessional* adj3 journal*).tw,kw,kf. (4)

98 (unprofessional* adj3 periodical*).tw,kw,kf. (0)

99 (unprofessional* adj3 publication?).tw,kw,kf. (3)

100 (unprofessional* adj3 publish*).tw,kw,kf. (1)

101 (untrust* adj3 edit*).tw,kw,kf. (0)

102 (untrust* adj3 journal*).tw,kw,kf. (0)

103 (untrust* adj3 periodical?).tw,kw,kf. (0)

104 (untrust* adj3 publication?).tw,kw,kf. (1)

105 (untrust* adj3 publish*).tw,kw,kf. (2)

106 pseudo-journal*.tw,kw,kf. (13)

107 pseudo-periodical*.tw,kw,kf. (5)

108 pseudo-publish*.tw,kw,kf. (2)

109 Beall* list.tw,kw,kf. (46)

110 or/1-109 (1564)

111 limit 110 to yr="2012-current" (1112)

112 (comment or editorial or news or newspaper article).pt. (1853727)

113 111 not 112 [OPINION PIECES REMOVED] (869)

114 remove duplicates from 113 (586)

115 114 use medall (333)

116 114 use emczd (156)

117 114 use eric (16)

118 114 not (115 or 116 or 117) (81)
