# Supplementary material for: Checklists to detect potential predatory biomedical journals: a systematic review
Source: BMC Med. 2020 May 18;18:104. doi: 10.1186/s12916-020-01566-1 (PMC7203891; doi:10.1186/s12916-020-01566-1)
Supplement: Supplementary file 1 — Additional file 1. Peer Review of Electronic Search Strategies (PRESS) Checklist. [file 12916_2020_1566_MOESM1_ESM.docx]

# *PRESS Guideline* 2015— Search Submission & Peer Review Assessment

Reference: McGowan J, Sampson M, Salzwedel DM, Cogo E, Foerster V, Lefebvre C. PRESS Peer Review of Electronic Search Strategies: 2015 guideline statement. *J Clin Epidemiol* 2016;75:40-6. Available: <http://www.jclinepi.com/article/S0895-4356(16)00058-5/pdf>.

**Search submission: This section to be filled in by the searcher**

Searcher: Becky Skidmore	Email: [bskidmore@rogers.com](mailto:bskidmore@rogers.com)

Date submitted:	7 Nov 2018	Date requested by: 10 Nov 2018 AM

| 1. **Systematic Review Title** |  |
| --- | --- |

Systematic Review of Checklists to Detect Potential Predatory Biomedical Journals and Publishers

2. **This search strategy is …**

| X | My PRIMARY (core) database strategy — First time submitting a strategy for search question and database |
| --- | --- |
|  | My PRIMARY (core) strategy — Follow-up review NOT the first time submitting a strategy for search question and database. If this is a response to peer review, itemize the changes made to the review suggestions |
|  | SECONDARY search strategy— First time submitting a strategy for search question and database |
|  | SECONDARY search strategy — NOT the first time submitting a strategy for search question and database. If  this is a response to peer review, itemize the changes made to the review suggestions |

3. **Database** (e.g., MEDLINE, CINAHL)	*[mandatory]*

MEDLINE

4. **Interface** (e.g., Ovid, EbscoHost…)	*[mandatory]*

Ovid

5. **Research Question** (Describe the purpose of the search)	*[mandatory]*

6. **PICO Format** Outline the PICOs for your question — i.e., Patient, Intervention, Comparison, Outcome, and Study Design — as applicable

| **P** | Predatory Journals |
| --- | --- |
| **I / Exposure** | Checklists to detect a potentially predatory journal or publisher |
| **C** |  |
| **O** |  |
| **S** |  |

7. **Inclusion Criteria** (List criteria such as age groups, study designs, etc., to be included) *[optional]*
8.

**This search strategy is …**

Publication years 2012-current

9. **Exclusion Criteria** (List criteria such as study designs, date limits, etc., to be excluded) **[optional]**

Opinion pieces and editorials

10. **Was a search filter applied?** No

**If YES, which one(s) (e.g., Cochrane RCT filter, PubMed Clinical Queries filter)? Provide the source if this is a published filter.** *[mandatory if YES to previous question* — *textbox]*

11. **Notes or comments you feel would be useful for the peer reviewer**	*[optional]*

It is possible info will not be restricted to biomedical field so terminology and choice of databases have been adjusted for this possible broader scope (e.g., also including library science databases, Web of Science)

Have shown Ovid multifile search instead of just MEDLINE

“Series” was suggested as a possible synonym for checklists but was tested and discarded

Much of the vocabulary pertaining to predatory journals has been previously PRESSed (dark and rogue have been added)

There will be an extensive follow-up grey literature search of library web sites, YouTube, etc.

Volume in published literature is very small – one option is to only search on the population (predatory journals) for the electronic database searches (684 records after removing duplicates in Ovid). Thoughts?

12. **Please copy and paste your search strategy here, exactly as run, including the number of hits per line. [mandatory]**

Database: Embase Classic+Embase <1947 to 2018 November 06>, Ovid MEDLINE(R) ALL <1946 to November 06, 2018>, PsycINFO <1806 to October Week 5 2018>, ERIC <1965 to August 2018>

Search Strategy:

--------------------------------------------------------------------------------

1	(predator* adj3 edit*).tw,kw,kf. (29)

2	(predator* adj3 journal*).tw,kw,kf. (393)

3	(predator* adj3 periodical?).tw,kw,kf. (6)

4	(predator* adj3 publication?).tw,kw,kf. (48)

5	(predator* adj3 publish*).tw,kw,kf. (373)

6	(bogus adj3 edit*).tw,kw,kf. (2)

7	(bogus adj3 journal*).tw,kw,kf. (7)

8	(bogus adj3 periodical?).tw,kw,kf. (0)

9	(bogus adj3 publication?).tw,kw,kf. (0)

10	(bogus adj3 publish*).tw,kw,kf. (1)

11	(dark adj3 edit*).tw,kw,kf. (32)

12	(dark adj3 journal*).tw,kw,kf. (9)

13	(dark adj3 periodical?).tw,kw,kf. (4)

14	(dark adj3 publication?).tw,kw,kf. (2)

15	(dark adj3 publish*).tw,kw,kf. (19)

16	(decepti* adj3 edit*).tw,kw,kf. (21)

17	(decepti* adj3 journal*).tw,kw,kf. (15)

18	(decepti* adj3 periodical?).tw,kw,kf. (0)

19	(decepti* adj3 publication?).tw,kw,kf. (3)

20	(decepti* adj3 publish*).tw,kw,kf. (20)

21	(disreput* adj3 edit*).tw,kw,kf. (0)

22	(disreput* adj3 journal*).tw,kw,kf. (3)

23	(disreput* adj3 periodical?).tw,kw,kf. (0)

24	(disreput* adj3 publication?).tw,kw,kf. (3)

25	(disreput* adj3 publish*).tw,kw,kf. (0)

26	(distrust* adj3 edit*).tw,kw,kf. (1)

27	(distrust* adj3 journal*).tw,kw,kf. (2)

28	(distrust* adj3 periodical?).tw,kw,kf. (0)

29	(distrust* adj3 publication?).tw,kw,kf. (0)

30	(distrust* adj3 publish*).tw,kw,kf. (5)

31	(exploit* adj3 edit*).tw,kw,kf. (107)

32	(exploit* adj3 journal*).tw,kw,kf. (29)

33	(exploit* adj3 periodical?).tw,kw,kf. (1)

34	(exploit* adj3 publication?).tw,kw,kf. (37)

35	(exploit* adj3 publish*).tw,kw,kf. (93)

36	(fake? adj3 edit*).tw,kw,kf. (11)

37	(fake? adj3 journal*).tw,kw,kf. (36)

38	(fake? adj3 periodical?).tw,kw,kf. (0)

39	(fake? adj3 publication?).tw,kw,kf. (4)

40	(fake? adj3 publish*).tw,kw,kf. (20)

41	(hoax$2 adj3 edit*).tw,kw,kf. (1)

42	(hoax$2 adj3 journal*).tw,kw,kf. (5)

43	(hoax$2 adj3 periodical?).tw,kw,kf. (0)

44	(hoax$2 adj3 publication?).tw,kw,kf. (2)

45	(hoax$2 adj3 publish*).tw,kw,kf. (4)

46	(illegitim* adj3 edit*).tw,kw,kf. (3)

47	(illegitim* adj3 journal*).tw,kw,kf. (19)

48	(illegitim* adj3 periodical?).tw,kw,kf. (0)

49	(illegitim* adj3 publication?).tw,kw,kf. (6)

50	(illegitim* adj3 publish*).tw,kw,kf. (12)

51	(mislead* adj3 edit*).tw,kw,kf. (42)

52	(mislead* adj3 journal*).tw,kw,kf. (36)

53	(mislead* adj periodical?).tw,kw,kf. (0)

54	(mislead* adj3 publication?).tw,kw,kf. (57)

55	(mislead* adj publish*).tw,kw,kf. (5)

56	(non-legitim* adj3 edit*).tw,kw,kf. (0)

57	(non-legitim* adj3 journal*).tw,kw,kf. (0)

58	(non-legitim* adj3 periodical?).tw,kw,kf. (0)

59	(non-legitim* adj3 publication?).tw,kw,kf. (0)

60	(non-legitim* adj3 publish*).tw,kw,kf. (0)

61	(questionabl* adj3 edit*).tw,kw,kf. (24)

62	(questionabl* adj3 journal*).tw,kw,kf. (38)

63	(quesionabl* adj3 periodical?).tw,kw,kf. (0)

64	(questionabl* adj3 publication?).tw,kw,kf. (44)

65	(questionabl* adj3 publish*).tw,kw,kf. (48)

66	(racket? adj3 edit*).tw,kw,kf. (0)

67	(racket? adj3 journal*).tw,kw,kf. (1)

68	(racket? adj3 periodical?).tw,kw,kf. (0)

69	(racket? adj3 publication?).tw,kw,kf. (0)

70	(racket? adj3 publish*).tw,kw,kf. (0)

71	(rogue adj3 edit*).tw,kw,kf. (4)

72	(rogue adj3 journal*).tw,kw,kf. (2)

73	(rogue adj3 periodical?).tw,kw,kf. (0)

74	(rogue adj3 publication?).tw,kw,kf. (0)

75	(rogue adj3 publish*).tw,kw,kf. (4)

76	(scam* adj3 edit*).tw,kw,kf. (3)

77	(scam* adj3 journal*).tw,kw,kf. (9)

78	(scam* adj3 periodical?).tw,kw,kf. (0)

79	(scam* adj3 publication?).tw,kw,kf. (0)

80	(scam* adj3 publish*).tw,kw,kf. (5)

81	(sham adj3 edit*).tw,kw,kf. (1)

82	(sham adj3 journal*).tw,kw,kf. (9)

83	(sham adj3 periodical?).tw,kw,kf. (0)

84	(sham adj3 publication?).tw,kw,kf. (1)

85	(sham adj3 publish*).tw,kw,kf. (50)

86	(spam* adj3 edit*).tw,kw,kf. (1)

87	(spam* adj3 journal*).tw,kw,kf. (4)

88	(spam* adj3 periodical?).tw,kw,kf. (0)

89	(spam* adj3 publication?).tw,kw,kf. (2)

90	(spam* adj3 publish*).tw,kw,kf. (5)

91	(unethic* adj3 edit*).tw,kw,kf. (21)

92	(unethic* adj3 journal*).tw,kw,kf. (22)

93	(unethic* adj3 periodical?).tw,kw,kf. (0)

94	(unethic* adj3 publication?).tw,kw,kf. (52)

95	(unethic* adj3 publish*).tw,kw,kf. (51)

96	(unprofessional* adj3 edit*).tw,kw,kf. (1)

97	(unprofessional* adj3 journal*).tw,kw,kf. (4)

98	(unprofessional* adj3 periodical*).tw,kw,kf. (0)

99	(unprofessional* adj3 publication?).tw,kw,kf. (3)

100	(unprofessional* adj3 publish*).tw,kw,kf. (1)

101	(untrust* adj3 edit*).tw,kw,kf. (0)

102	(untrust* adj3 journal*).tw,kw,kf. (0)

103	(untrust* adj3 periodical?).tw,kw,kf. (0)

104	(untrust* adj3 publication?).tw,kw,kf. (1)

105	(untrust* adj3 publish*).tw,kw,kf. (2)

106	pseudo-journal*.tw,kw,kf. (13)

107	pseudo-periodical*.tw,kw,kf. (5)

108	pseudo-publish*.tw,kw,kf. (2)

109	Beall* list.tw,kw,kf. (44)

110	or/1-109 (1553)

111	limit 110 to yr="2012-current" (1101)

112	Checklist/ use emczd,medall (24630)

113	Check Lists/ use eric (6639)

114	Editorial Policies/ use medall (7197)

115	guideline.pt. (16004)

116	Guidelines/ use eric (23366)

117	Guides/ use eric (8271)

118	exp Journalism/st use medall (998)

119	Open Access Publishing/st use medall (36)

120	exp Peer Review/st use medall (2244)

121	Publishing/st use medall (5272)

122	checklist*.tw,kw,kf. (117991)

123	check list*.tw,kw,kf. (19096)

124	guide*.tw,kw,kf. (1713265)

125	guidance*.tw,kw,kf. (319738)

126	criteria.tw,kw,kf. (1516630)

127	criterion.tw,kw,kf. (228194)

128	(tool or tools).tw,kw,kf. (1634450)

129	(instrument or instruments).tw,kw,kf. (564019)

130	algorithm?.tw,kw,kf. (504760)

131	instruction?.tw,kf,kw. (545248)

132	(inventory or inventories).tw,kf,kw. (308435)

133	(list or lists or listing or listings).tw,kf,kw. (461420)

134	primer?.tw,kw,kf. (204194)

135	or/112-134 [CHECKLISTS] (6958828)

136	111 and 135 [PREDATORY JNL CHECKLISTS] (341)

137	(comment or editorial or news or newspaper article).pt. (1850264)

138	136 not 137 [OPINION PIECES REMOVED] (286)

139	remove duplicates from 138 (206)

***************************

**Peer review assessment: this section to be filled in by the reviewer**

|  | Reviewer: Kaitryn Campbell | Email: [kcamlolo668@gmail.com](mailto:kcamlolo668@gmail.com) | Date completed: 10 Nov 2018 |
| --- | --- | --- | --- |
|  |  |  |  |

Do you wish to be acknowledged? (If yes, the review team will be advised to add an acknowledgement to any publications related to this work). No – unless your organization requires it

The suggested acknowledgement is “ We thank Xxxxx Yyyyyy, MLIS, AHIP (xxxxx Health Sciences Library, University of xxxxxx) for peer review of the MEDLINE search strategy.” [please edit to indicate your name, postnomials and institutional affiliation as you would like them presented].

|  | **1. TRANSLATION** |  |  | | |
| --- | --- | --- | --- | --- | --- |
|  | |  | |  |  |
|  | | A -­‐No revisions | | X |  |
|  | | B -­‐ Revision(s) suggested | |  |  |
|  | | C -­‐ Revision(s) required | |  |  |

If “B” or “C,” please provide an explanation or example:

**2. BOOLEAN AND PROXIMITY OPERATORS**

| A -­‐No revisions | X |
| --- | --- |
| B -­‐ Revision(s) suggested |  |
| C -­‐ Revision(s) required |  |

If “B” or “C,” please provide an explanation or example:

**3. SUBJECT HEADINGS**

| A -­‐No revisions | X |
| --- | --- |
| B -­‐ Revision(s) suggested |  |
| C -­‐ Revision(s) required |  |

If “B” or “C,” please provide an explanation or example:

**4. TEXT WORD SEARCHING**

| A -­‐No revisions | X |
| --- | --- |
| B -­‐ Revision(s)suggested |  |
| C -­‐ Revision(s) required |  |

If “B” or “C,” please provide an explanation or example:

**5. SPELLING, SYNTAX, AND LINE NUMBERS**

| A -­‐No revisions | X |
| --- | --- |
| B -­‐ Revision(s)suggested |  |
| C -­‐ Revision(s) required |  |

If “B” or “C,” please provide an explanation or example:

**6. LIMITS AND FILTERS**

| A -­‐No revisions | X |
| --- | --- |
| B -­‐ Revision(s) suggested |  |
| C -­‐ Revision(s) required |  |

If “B” or “C,” please provide an explanation or example:

OVERALL EVALUATION (Note: If one or more “revision required” is noted above, the response below must be “revisions required”.)

| A -­‐No revisions | X |
| --- | --- |
| B -­‐ Revision(s) suggested |  |
| C -­‐ Revision(s) required |  |

Additional comments:

Solidly done. No errors or omissions found. Re: including just the “predatory journals” concept alone as the strategy for the bibliographic database searching, this seems like a strong option for 2 reasons: 1) limited retrieval numbers; 2) I have some experience doing “checklist” searches and found the description of these types of items can be extremely variable. My personal preference would be to leave the checklist concept out.
